# The Molecular Mechanism and Therapeutic Strategy of Cardiorenal Syndrome Type 3

**DOI:** 10.31083/j.rcm2402052

**Published:** 2023-02-06

**Authors:** Yong Liu, Xu Guan, Yuming Shao, Jie Zhou, Yinghui Huang

**Affiliations:** ^1^Department of Nephrology, The Key Laboratory for the Prevention and Treatment of Chronic Kidney Disease of Chongqing, Chongqing Clinical Research Center of Kidney and Urology Diseases, Xinqiao Hospital, Army Medical University (Third Military Medical University), 400037 Chongqing, China; ^2^Medical Division, Xinqiao Hospital, Army Medical University, 400037 Chongqing, China; ^3^Department of Oncology, Southwest Cancer Center, Southwest Hospital, Army Medical University, 400038 Chongqing, China

**Keywords:** CRS3, mitochondrial dysfunction, crosstalk, molecular mechanisms, therapeutic strategies

## Abstract

Cardiorenal syndrome type 3 (CRS3) is defined as acute kidney injury (AKI)-induced acute cardiac dysfunction, characterized by high morbidity and mortality. CRS3 often occurs in elderly patients with AKI who need intensive care. Approximately 70% of AKI patients develop into CRS3. CRS3 may also progress towards chronic kidney disease (CKD) and chronic cardiovascular disease (CVD). However, there is currently no effective treatment. Although the major intermediate factors that can mediate cardiac dysfunction remain elusive, recent studies have summarized the AKI biomarkers, identified direct mechanisms, including mitochondrial dysfunction, inflammation, oxidative stress, apoptosis and activation of the sympathetic nervous system (SNS) and renin-angiotensin-aldosterone system (RAAS), inflammasome, as well as indirect mechanisms such as fluid overload, electrolyte imbalances, acidemia and uremic toxins, which are involved in the pathophysiological changes of CRS3. This study reviews the main pathological characteristics, underlying molecular mechanisms, and potential therapeutic strategies of CRS3. Mitochondrial dysfunction and inflammatory factors have been identified as the key initiators and abnormal links between the impaired heart and kidney, which contribute to the formation of a vicious circle, ultimately accelerating the progression of CRS3. Therefore, targeting mitochondrial dysfunction, antioxidants, Klotho, melatonin, gene therapy, stem cells, exosomes, nanodrugs, intestinal microbiota and Traditional Chinese Medicine may serve as promising therapeutic approaches against CRS3.

## 1. Introduction 

**Fig. 1.**
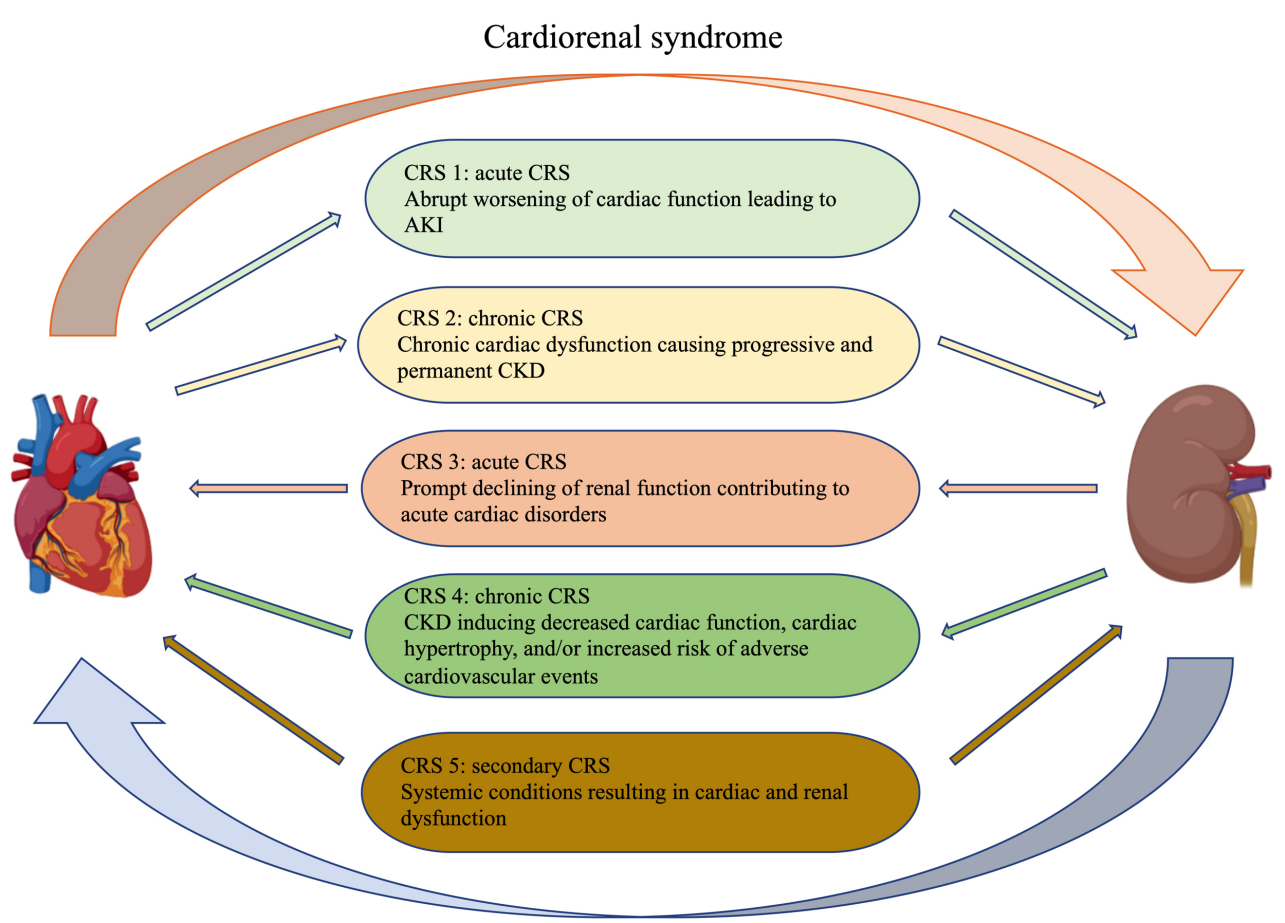
**Classification of cardiorenal syndrome**. The Figures in this review are constructed by the online website (https://app.biorender.com).

Physiological communication between the heart and kidney is essential to preserve metabolic waste removal, hemodynamic stability and bodily function [1]. However, in pathological states, an impaired organ often leads to the dysfunction of another organ. Cardiorenal syndrome (CRS) was used to describe this complex pathological interaction between the heart and kidney [2, 3, 4]. According to the primary or secondary organic dysfunction, CRS is divided into cardiorenal syndrome (type 1 and 2) and renal-cardiac syndrome (type 3 and 4). Depending on whether the primary organ dysfunction is acute (type 1 and 3) or chronic (type 2 and 4) at the time of onset [5]. In addition, CRS type 5 describes a systemic disease such as diabetes or sepsis that causes both cardiac and renal dysfunction [6] (Fig. 1). In fact, many patients may develop or transform between different CRS subtypes during their disease progression.

Cardiorenal syndrome type 3 (CRS3), also known as acute renal-cardiac syndrome, is an episode of acute cardiac dysfunction caused by acute kidney injury (AKI) [7]. The main clinical manifestations of CRS3 include acute heart failure, acute myocardial infarction, tachyarrhythmia and acute cardiogenic shock [4, 8]. It is generally accepted that AKI is the pathogenic factor and initiator of CRS3, and approximately 70% of AKI patients develop into CRS3 [9, 10]. AKI is also the result of a rapid deterioration of cardiac function in CRS type 1, as well as the cause of acute cardiac injury in CRS type 3. Therefore, the interplay between the damaged kidney and heart may form a vicious cycle, which further aggravates the development CRS3 [5]. Thus, timely and effective intervention is necessary to impede the progression of this disease. Although CRS3 has attracted increased attention in recent years, the pathophysiological and molecular mechanisms of CRS3 remain largely unknown.

In this review, the literature search strategy and search terms include AKI, Cardiorenal syndrome type 3, molecular mechanisms of CRS3, therapeutic strategies of CRS3 as well as targets of CRS3. We summarized the pathophysiological changes, pathogenesis, and underlying mechanisms of CRS3, and discussed the potential therapeutic targets for CRS3. We have summarized and classified the novel biomarkers of AKI caused by different injuries. These biomarkers will be beneficial for accurate diagnostic and prognostic evaluation of progressive AKI, which can guide the adoption of therapeutic management strategies. Moreover, these AKI biomarkers may also be key molecules in the interplay between the kidney and heart. Timely monitoring and interventions of these AKI biomarkers may also delay the progression of CRS3 (Table 1, Ref. [11, 12, 13, 14, 15, 16, 17, 18, 19, 20, 21, 22, 23, 24, 25, 26, 27, 28, 29, 30, 31, 32, 33, 34, 35, 36]).

**Table 1.** **Novel AKI biomarkers**.

Biomarker	Sample	Origin	References
TIMP-2	Urine	Distal tubule	[11, 12]
IGFBP7	Urine	Proximal tubule	[11, 13]
NGAL	Urine or Plasma	Distal tubule, Epithelial cells, Neutrophils	[14]
KIM-1	Urine	Proximal tubule	[15]
Collectrin↓	Urine	Proximal tubule	[16]
OLFM4	Urine	Neutrophils and Epithelial cells	[17]
L-FABP	Urine	Proximal tubule	[18]
CCL14	Urine	Tubular epithelial cells	[19]
Dickkopf-3	Urine	Tubular epithelial cells	[20]
IL-18	Urine	Multiple cell types	[21, 22]
Cd, Cu and Zn	Urine	—	[23]
MMP-9	Urine	Proximal tubule	[24]
α-1-microglobulin	Urine	Hepatocytes	[25]
α1-acid glycoprotein	Urine	Hepatocytes	[26]
Albumin	Urine or Serum	Hepatocytes	[27]
N-acetyl-β-d-Glucosaminidase	Urine	Proximal tubule	[28]
Calprotectin	Urine or Plasma	Neutrophils	[29, 30]
Cystatin C	Plasma or Serum	Nucleated cells	[31]
Lnc-HILPDA, Lnc-PRND	Serum	Kidney	[32]
suPAR	Plasma	Immune cells, Endothelial cells	[33, 34]
Proenkephalin A	Plasma	Multiple cell types	[35]
GDF15	Plasma	Proximal tubule	[36]

TIMP-2, tissue inhibitor of metalloproteinases 2; IGFBP7, insulin-like growth factor-binding protein 7; NGAL, neutrophil gelatinase-associated lipocalin; KIM-1, kidney injury molecule 1; OLFM4, Olfactomedin-4; L-FABP, liver-type fatty acid-binding protein; CCL14, C-C motif chemokine ligand 14; Dickkopf-3, Dickkopf-related protein 3; IL-18, interleukin-18; MMP-9, matrix metalloproteinase-9; Lnc-HILPDA, long non-coding RNA HILPDA; Lnc-PRND, long non-coding RNA PRND; suPAR, soluble urokinase plasminogen activator receptor; GDF-15, growth/differentiation factor 15; ↓ represents a reduced level.

## 2. Molecular Mechanisms of CRS3 

Although the precise pathophysiological mechanisms of CRS3 remain unclear, AKI is considered to possess direct and indirect mechanisms on cardiac structure and function. Direct mechanisms can be attributed to mitochondrial dysfunction, inflammation, oxidative stress, apoptosis, activation of the sympathetic nervous system (SNS), renin-angiotensin-aldosterone system (RAAS) and inflammasome. Indirect mechanisms include fluid overload, electrolyte imbalances, acidemia and uremic toxins [4, 37].

### 2.1 Direct Mechanisms

#### 2.1.1 Mitochondrial Dysfunction

**Fig. 2.**
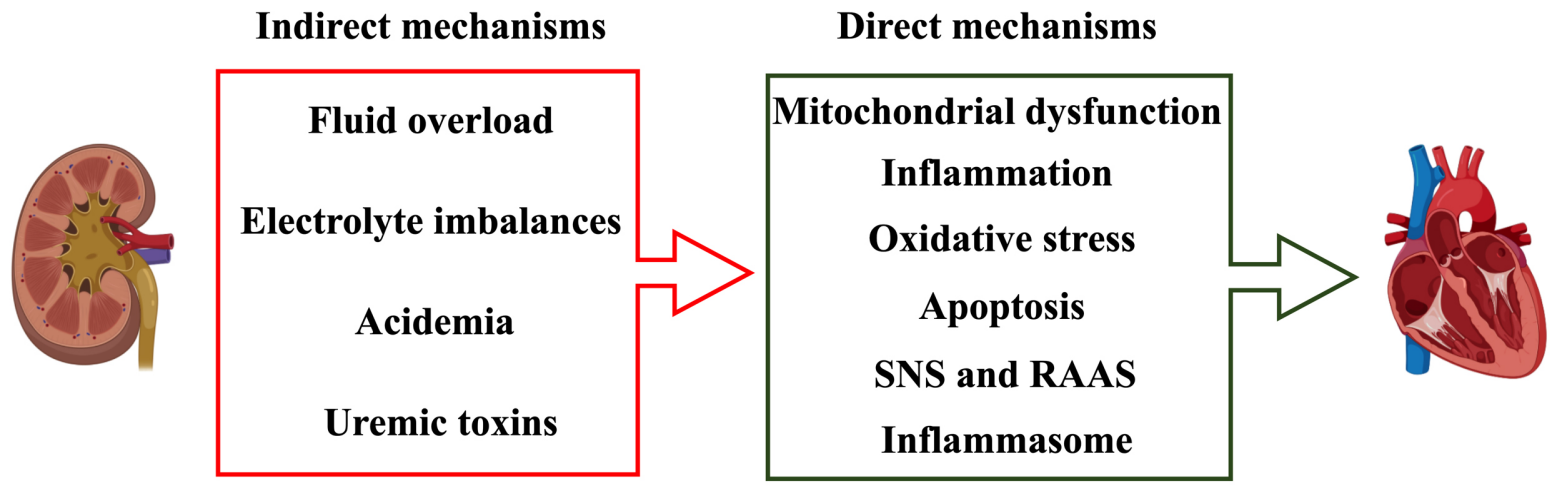
**Direct and indirect mechanisms contributing to CRS3**.

Recent studies have demonstrated that mitochondrial dysfunction as an important contributor to myocardial injury in CRS3 [7]. Therefore, maintaining mitochondrial homeostasis is a promising strategy for the treatment of CRS3 [7, 38]. Both mitochondrial unfolded protein response (${\mathrm{UPR}}^{\text{mt}}$) and mitophagy are protective procedures to maintain mitochondrial quality control [39, 40]. Mitophagy is activated after mitochondrial injury to remove damaged mitochondria via a lysosome-mediated organelle degradation mechanism [41, 42]. Previous studies have shown that mitophagy can reduce mitochondrial oxidative stress, suppress the inflammatory response, inhibit mitochondria-dependent cardiomyocyte apoptosis, reverse mitochondrial-involved energy metabolism, enhance mitochondrial antioxidant capacity, promote mitochondrial membrane potential stability, and maintain intracellular calcium homeostasis [42, 43, 44]. In addition, the ${\mathrm{UPR}}^{\text{mt}}$ is activated to maintain mitochondrial membrane integrity by regulating the expression of mitochondria-related proteins. Previous studies have shown that mitophagy and ${\mathrm{UPR}}^{\text{mt}}$ exert protective roles in a variety of cardiovascular diseases (CVDs), including myocardial infarction and myocardial ischemia/reperfusion injury (IRI) [45, 46]. More importantly, numerous studies have shown that mitophagy and ${\mathrm{UPR}}^{\text{mt}}$ also play protective roles in renal diseases such as AKI [47, 48]. Therefore, targeting mitochondrial dysfunction to maintain mitochondrial homeostasis may not only improve AKI but also ameliorate AKI-related myocardial injury (Fig. 2).

#### 2.1.2 Inflammation

Inflammatory response is an important pathological process and therapeutic target of CRS [49, 50]. It has been observed that the expressions of several inflammatory factors are significantly altered after IRI in the unilateral kidney, including tumor necrosis factor-a (TNF-α), interleukin-1β (IL-1β), interleukin-6 (IL-6), interleukin-17 (IL-17) and interleukin-12 (IL-12), which are also elevated in myocardial hypertrophy [51, 52]. Previous studies reported inflammatory factors, including IL-6, IL-10 and TNF-α, could cause cardiovascular endothelial damage, leading to cardiac hypertrophy, fibrosis, and atherosclerosis [53, 54, 55, 56]. Kelly *et al*. [57] found that anti-TNF-α treatment reversed AKI-induced cardiac injury. Wang *et al*. [10] showed that AKI promoted inflammatory reaction, and abnormally elevated serum IL-6 concentration, which upregulated the expression of growth factor receptor-binding protein 2 (Grb2). Grb2 mediated the disorders of myocardial cell metabolism and ATP production by affecting the AKT kinase/mammalian target of rapamycin (Akt/mTOR) signaling pathway, impairing myocardial relaxation, and aggravating the cardiac injury after AKI [10, 58]. Inflammatory factors may be an important mediator of interaction between the kidney and heart. Targeting the inflammatory response and inhibition of the release and circulation of inflammatory factors may be a potential therapeutic strategy.

#### 2.1.3 Oxidative Stress

Accumulating evidence has demonstrated that oxidative stress is closely related to the dysfunction of cellular components that can lead to organ disorders, and is recognized as one of the major causes of IRI-induced distant organ injury [59, 60, 61]. Oxidative stress is also considered to be a direct by-product of mitochondrial damage in myocardial cells involved in the pathogenesis of CRS3 [62]. Oxidative stress increases the channels of calcium release, such as the inositol 1,4,5-triphosphate receptor, and inhibits the activity of calcium reuptake proteins, including sarcoplasmic/endoplasmic reticulum calcium ATPase 2a [63]. This results in a systolic calcium deficit and/or diastolic calcium leak, which increases the possibility of arrhythmogenic events and diminishes cardiac contractile capacity [64]. Eventually, impaired mitochondria provoke cell death by activating caspase 3/7/9 in myocardial cells during CRS3 [65]. The death of myocardial cells then prompts the inflammatory response and the release of inflammatory factors, further aggravating the damage of myocardial cells in the process of CRS3 [10]. Caio-Silva *et al*. [62] used a unilateral renal IRI mouse model to evaluate oxidative stress and antioxidant parameters of the kidney and heart. The results showed that the activities of antioxidant enzymes superoxide dismutase (SOD) in kidney tissues significantly increased, and the bioavailability of nitric oxide (NO) was also increased [62]. The activities of nicotinamide adenine dinucleotide phosphate (NADPH) oxidase (NOX) and nitric oxide synthase (NOS) in myocardial tissues were enhanced, accompanied by the aggravation of cell damage, 8 days after renal IRI. Cai *et al*. [65] reported in a CRS3 mouse model that CRS3 resulted in lower heart function, increased inflammatory responses and exacerbated myocardial oxidative stress than in sham mice. Mitochondrial oxidative stress and the inflammation response have been proposed to reduce cardiomyocyte viability and function during CRS3. Neres-Santos *et al*. [7] reported that vitamin C, an antioxidant, exerted renocardiac-protective effects by reducing NO levels and inducible nitric oxide synthase (iNOS) expression in the kidney and heart. These studies suggest that oxidative stress is a direct mechanism involved in the pathogenesis of CRS3.

#### 2.1.4 Apoptosis

Various types of cell death have been reported to be involved in acute renal and cardiac injury, including apoptosis, necrosis, pyroptosis, ferroptosis and autophagy [66, 67, 68, 69, 70, 71, 72, 73]. Apoptosis is one of the most common modes of cell death, which is activated by death receptors [74]. Kelly *et al*. [57] first reported that AKI leads to an upregulated expression of intercellular cell adhesion molecule-1 (ICAM-1) and a significant increase in peroxidase activity and apoptosis in cardiomyocytes, accompanied by a decrease in left ventricular dilatation and short-axis shortening of the left ventricle. This study found that the inflammatory factors, TNF-α and IL-1β were significantly increased after renal ischemia, and that TNF-α neutralizing antibody treatment could reverse cardiomyocyte apoptosis [57]. The study also demonstrated that decreased cardiac function may be an important determinant of increased mortality in AKI patients. In addition, in an AKI rat model, a significant increase in myocardial apoptosis was also observed [75, 76]. These studies suggest that cardiomyocyte apoptosis may be associated with renal injury, and that excessive reactive oxygen species (ROS) and inflammatory factors may be involved.

#### 2.1.5 SNS and RAAS

AKI is accompanied by continuous activation of the sympathetic nervous system (SNS) and the renin-angiotensin-aldosterone system (RAAS) [56, 77, 78, 79]. The activated SNS will induce the release of neuropeptide Y, which is responsible for the formation of neointima, vasoconstriction and impairment of the immune system [80]. SNS activation has multiple direct negative effects on the heart, including increasing myocardial oxygen demand, destroying calcium homeostasis, promoting cardiomyocyte apoptosis and hypertrophy [81, 82, 83]. In addition, SNS can further activate RAAS. Studies have shown that RAAS activation has become a major risk factor for AKI. RAAS plays a vital role in regulating renal hemodynamics, function and pathophysiology during kidney diseases [84, 85]. Recent studies have demonstrated that urinary renin, the rate limiting enzyme of RAAS, is an indicator of renal RAAS activity, and the increase of renin is related to the severity of AKI [78, 86]. RAAS activation leads to an increase in angiotensin II, which results in systemic vasoconstriction and extracellular volume expansion by increasing sodium retention [87], leading to cardiac remodeling and ventricular hypertrophy [88]. *In vitro* studies have shown that angiotensin II can induce hypertrophy, cell reprogramming and necrosis of cardiomyocytes, and cardiac fibrosis [89, 90, 91]. Although the role of RAAS and SNS in CRS3 still lacks solid evidence, further in-depth experimental studies may help to elucidate the precise effects and underlying mechanisms.

#### 2.1.6 Inflammasome

Inflammasome, a multiprotein complex which can trigger the cleavage and activation of proinflammatory cytokine including IL-1β and IL-18, plays an important role in the innate immune response. Previous studies have shown that the activation of inflammasome promotes AKI, whereas inhibition of inflammasome impedes the progression of AKI. In addition, some small molecules and compounds have been found to improve AKI by inhibiting the activation of inflammasome [92, 93]. Song *et al*. [94] demonstrated that astaxanthin alleviates contrast-induced AKI through the reactive oxygen species/nod-like receptor pyrin domain-containing protein 3 (ROS/NLRP3) inflammasome. Huang *et al*. [95] found that disulfiram alleviated AKI induced by lipopolysaccharide by inhibiting oxidative stress and NLRP3 inflammasome activation. Maadawy, Walaa H *et al*. [96] found that 6-Paradol alleviates diclofenac induced AKI by regulating autophagy enhancement mediated by AMP-activated protein kinases/AKT kinase/mammalian target of rapamycin (AMPK/AKT/mTOR) and the NLRP3 inflammasome pathway. Li *et al*. [97] found that spermidine improved AKI by inhibiting the activation of the NLRP3 mediated-inflammasome in macrophages. Collectively, these studies provide evidence that inhibition of inflammasome activation could improve AKI. Simultaneously, inflammasome activation can also promote myocardial cell damage, including cardiac hypertrophy, fibrosis and myocardial proptosis [98, 99]. Blocking the activation of inflammasome can reduce the hospitalization rate and mortality of patients with heart failure [100]. Current evidence shows that inflammasome are important mediators of AKI and cardiac inflammation and are a promising therapeutic target [101]. Yamaguchi *et al*. [102] found that Indoxyl Sulfate activated NLRP3 inflammasome induces myocardial fibrosis and cardiac systolic dysfunction associated with hypertrophy. Trentin-Sonoda *et al*. [103] showed that Caspase-1 is the key molecule of cardiac remodeling during cardiorenal syndrome type 3 in the mouse model. Caspase-1 deficiency can result in cardiac hypertrophy in a renal ischemia-reperfusion mouse model. Therefore, inflammasome inhibition is a potential therapeutic target for AKI and CRS3. Table 2 (Ref. [104, 105, 106, 107, 108, 109, 110, 111, 112, 113]) summarizes the clinical data related to the inflammasome biomarker in AKI and CRS patients.

**Table 2.** **Inflammasome component involvement in human AKI and CRS**.

Inflammasome component	Samples	Outcomes	References
NLRP3	Serum samples	NLRP3 in the septic shock group was significantly higher than that in the healthy control group.	Huang *et al*., 2022 [104]
IL-1β	Serum samples	IL-1β levels were augmented in septic AKI patients.	Zheng *et al*., 2021 [105]
			Shi *et al*., 2021 [106]
IL-1β	Serum samples	IL-1β levels were increased in serum of children undergoing congenital heart surgery.	Yang *et al*., 2021 [107]
IL-1β	Plasma samples	IL-1β levels were elevated in the CRS Type 5 group compared with healthy control subjects.	Brocca* et al*., 2015 [108]
IL-18	Plasma samples	IL-18 were significantly elevated in CRS.	Pastori *et al*., 2015 [109]
IL-18	Urine samples	Urine IL-18 were independently associated with AKI stage.	Duff *et al*., 2021 [110]
Caspase1, IL-18	Urine samples	IL-18 and caspase-1 were increased in patients undergoing coronary angiography.	Lau *et al*., 2018 [111]
NLRP6	Renal tubules	NLRP6 was reduced during human kidney injury.	Valiño-Rivas *et al*., 2020 [112]
ASC, Active-Caspase1	Renal interstitium	ASC and Active-Caspase1 were significantly increased in the RIAKI case compared to a healthy control.	Grivei *et al*., 2020 [113]

NLRP3, Nod-like receptor pyrin domain-containing protein 3; IL-1β, interleukin-1β; IL-18, interleukin-18; NLRP6, Nod-like receptor pyrin domain-containing protein 6; ASC, apoptosis-associated speck-like protein containing a caspase-recruitment domain; RIAKI, Rhabdomyolysis-induced acute kidney injury.

### 2.2 Indirect Mechanisms

Fluid overload, electrolyte imbalances, acidemia, and uremic toxins contribute to CRS3 under pathophysiological conditions. Fluid overload will lead to physiological abnormalities in multiple organs, especially in AKI patients [114, 115]. There is a time correlation between volume overload and the development of ventricular arrhythmias [116], since fluid overload increases the work of the heart, contributing to arrhythmias [117]. Therefore, complex arrhythmias have become a serious complication of AKI-induced cardiac injury and myocardial dysfunction. In turn, arrhythmias also increase the risk of renal failure [118].

Hyperkalemia is a common complication of severe AKI. Hyponatremia can cause premature atrial and ventricular contractions, while severe hypokalemia can cause prolongation of the Q-T interval, leading to ventricular tachycardia, ventricular fibrillation and cardiac arrest [119]. Hypernatremia will affect the heart during severe dehydration, and can result in tachycardia, decreased blood pressure, intracranial hemorrhage, and edema.

Metabolic acidosis is a common complication of AKI and a common indication for initiation of renal replacement therapy (RRT) [120], since elevated plasma hydrogen ion concentrations in patients with metabolic acidosis can seriously affect cardiac function [121, 122, 123]. Severe acidosis results in a marked decrease in cardiac contractility, which can be significantly improved by correcting acidosis [124, 125].

AKI causes an acute uremic state, as evidenced by electrolyte disturbances, disrupted volume stability, as well as the accumulation of metabolic toxins, including small water-soluble compounds, large intermediate molecules, and protein-bound uremic toxins (PBUTs) [126], which have been extensively investigated in chronic kidney disease (CKD); particularly indoxyl sulfate (IS), P-cresol sulfate (PCS), and indole-3-acetic acid (IAA) [127, 128, 129, 130]. Multiple studies have shown that IS promotes cardiac fibrosis and hypertrophy by inducing oxidative stress and inflammation. Similar to IS, PCS is toxic to blood vessels and the heart [131]. Huang *et al*. [132] demonstrated the toxic effect of PCS on cardiomyocytes by reducing cardiomyocyte proliferation and inducing mitochondrial damage. Lekawanvijit *et al*. [133] confirmed its deleterious effect on vascular reactivity in an *in vitro* model of an aortic ring exposed to PCS.

These accumulated metabolic toxins can result in cellular and tissue damage to the kidney and heart, while renal dysfunction increases the accumulation of uremic toxins, ultimately leading to the progression of CRS3. Although acute uremia in AKI may contribute to the cardiotoxicity of CRS3, further studies are required to determine their complex roles and underlying mechanisms in cardiotoxicity after AKI [134] (Fig. 2). Several studies, including our own, indicate that uremic toxins are closely related to CVDs [135, 136].

## 3. Therapeutic Strategies for CRS3

**Fig. 3.**
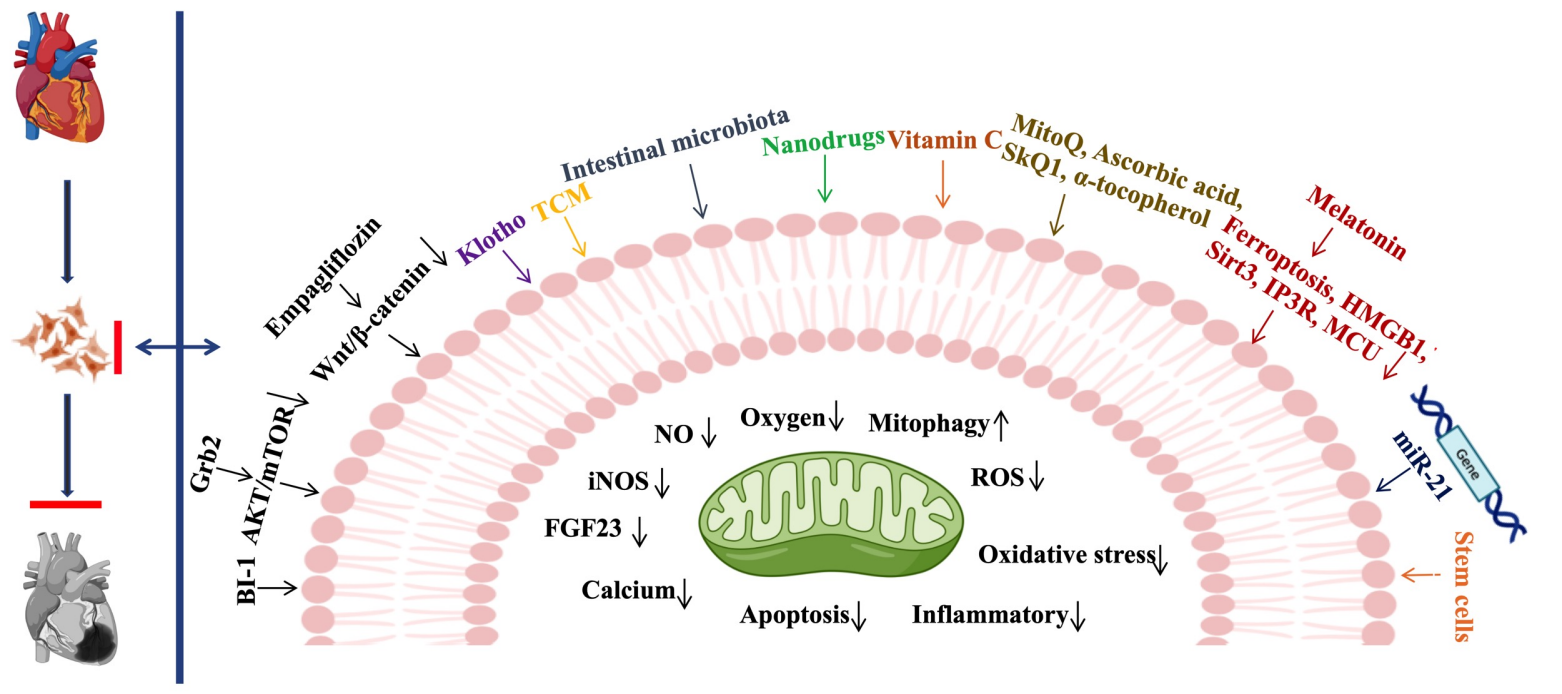
** Therapeutic strategies for CRS3**. BI- 1, Bax inhibitor-1; Grb2, Growth factor receptor-binding protein 2; AKT, AKT Kinase; mTOR, Mammalian target of rapamycin; TCM, Traditional Chinese Medicine; Wnt, Wingless/Integrated; MitoQ, Mitoquinone; SKQ1, 10-(6′-plastoquinonyl) decyltriphenyl phosphonium; HMGB1, high mobility group box 1; Sirt3, sirtuin-3; IP3R, Inositol 1,4,5-trisphosphate receptor; MCU, Mitochondrial calcium uniporter; NO, nitric oxide; iNOS, inducible nitric oxide synthase; FGF23, Fibroblast growth factor 23; ROS, reactive oxygen species.

The treatment of CRS3 is a difficult clinical challenge, since drugs used to treat CVD may possess potential nephrotoxicity, while treatment for AKI usually provokes myocardial damage. Therefore, targeted therapies to ameliorate AKI-related cardiac dysfunction are urgently needed. Since the pathophysiologic mechanism of CRS3 remains largely unknown, it is necessary to comprehensively study its molecular mechanisms and develop novel therapeutic targets for CRS3. In Fig. 3 we summarize the recently reported potential treatments targeting CRS3 .

### 3.1 Targeting Mitochondrial Dysfunction

Emerging evidence confirms that Bax inhibitor-1 (BI-1) could ameliorate myocardial injury in patients with CRS3 by activating mitochondrial UPR and FUN14 domain-containing protein 1 (FUNDC1) -mediated mitophagy, suggesting that BI-1 plays a crucial role in CRS3 [39]. Wang *et al*. [10] performed a proteomic analysis of CRS3 and identified Grb2 as an important regulator involved in AKI-related myocardial injury. Elevated Grb2 contributed to diastolic dysfunction and mitochondrial bioenergetics impairment, while the application of Grb2-specific inhibitor reversed these pathological changes during AKI. Abnormally elevated levels of Grb2 promotes mitochondrial metabolic disorder of myocardial cells by inhibiting the Akt/mTOR signaling pathway, which can lead to cardiac dysfunction [10]. Cai *et al*. [65] reported that empagliflozin can preserve mitochondrial structure, stabilize cardiomyocyte structure, maintain cardiac systolic and diastolic function, and reduce myocardial inflammation via activating the Wingless/integrated (Wnt)/β-catenin/FUNDC1-dependent mitophagy, suggesting that empagliflozin confers cardio-protection from AKI and can be used in the clinical treatment of cardiac dysfunction after AKI.

Collectively, these findings suggest that mitochondrial dysfunction is a common pathological feature and molecular mechanism of AKI and its related to cardiac injury. Targeting mitochondrial dysfunction and maintenance of mitochondrial homeostasis are promising strategies for the treatment of CRS3. However, the upstream mechanisms regulating mitophagy in CRS3 remain incompletely defined and further mechanistic studies are required.

### 3.2 Antioxidants

Accumulating evidence suggests that vitamin C confers renal and cardioprotective roles in CRS3. Recent studies have shown that vitamin C can not only improve AKI, but also protect the heart after AKI [7, 137]. It has been reported that vitamin C treatment can preserve kidney weight, restore renal function, reduce NO levels and iNOS expression, and improve oxygen consumption. After vitamin C treatment, oxygen consumption and NO levels were improved, oxidative stress was attenuated, mitochondrial damage was ameliorated, and myocardial cell damage was reduced. This study also showed that when the kidney was injured, vitamin C should be given as soon as possible to protect the kidney and heart from IRI [7]. However, this study also has some limitations. It does not clarify the mechanism by which ROS derived from the kidney affects cardiac damage and the role of vitamin C in the crosstalk between the kidney and the heart. In addition, several potential therapeutic approaches, such as Mitoquinone (MitoQ), ascorbic acid, 10-(6′-plastoquinonyl) decyltriphenyl phosphonium (SkQ1), and α-tocopherol, are reported to modulate mitochondrial ROS production in models of kidney and heart disease [138]. These antioxidants not only have been shown to improve renal function, but they can also improve cardiac function.

### 3.3 Klotho

Klotho is an anti-aging protein, predominantly expressed in the kidney. Previous studies have confirmed that Klotho exerts a protective role in AKI and CVD [139, 140]. Klotho deficiency not only aggravates AKI, promotes the transition of AKI to CKD, but also is closely related to CVD, suggesting that regulating endogenous or exogenous Klotho can provide renal and cardiac protection [141]. Another study has confirmed that Klotho has cardioprotective effect on CRS3 induced by renal IRI, mainly by preventing cardiac hypertrophy and ${\mathrm{Ca}}^{2+}$ circulatory dysfunction. This study also showed that Klotho acts on CRS by systematically preventing inflammation and inhibiting the abnormal increase of FGF23 in plasma, reducing adverse cardiac outcomes [51]. In addition, Klotho also has a protective effect in other types of CRS [134, 142]. It has been reported that the FGF23-Klotho axis is an important mediator of CRS and a potential therapeutic target [51].

### 3.4 Melatonin

Previous studies have confirmed that melatonin plays a protective role in AKI. Melatonin reduces AKI by inhibition of nuclear factor erythroid 2-related factor 2/solute carrier family 7 member 11 (Nrf2/Slc7a11) axis-mediated ferroptosis [143]. Melatonin significantly decreases folic acid (FA) induced AKI injury by inhibiting the nuclear translocation of high mobility group protein B1 (*HMGB1*) in renal tubular epithelial cells [144]. In addition, melatonin alleviates contrast-induced kidney injury by activating Sirt3 [145]. Melatonin also has cardioprotective effects, in cardiac IRI [146], septic cardiomyopathy [147], and drug-induced cardiotoxicity [148]. These results strongly suggest that melatonin may serve as a potential therapeutic treatment for CRS3. Wang *et al*. [9] confirmed that melatonin protects cardiac function from CRS3 by inhibiting inositol 1,4,5-trisphosphate receptor-mitochondrial calcium uniporter (IP3R-MCU) signaling. Melatonin preconditioning attenuates renal IRI-induced cardiac injury by maintaining myocardial diastolic function and reducing cardiomyocyte death. Melatonin can also enhance the effects of other treatments or medications for CRS. For example, the combination of melatonin and Exendin-4 has a protective effect on the heart and kidney of rats with CRS [149]. It was also reported that melatonin enhanced the therapeutic effect of mesenchymal stem cell-derived exosomes on renal IRI in rats [150].

### 3.5 Gene Therapy

microRNAs (miRNAs) are associated with the development and progression of various injuries, including renal and cardiac diseases, and CRS [151, 152]. Therefore, miRNAs are commonly used as disease biomarkers and potential therapeutic targets. Recent studies have shown that multiple miRNAs are also closely related to CRS [153]. It was reported that miR-21 is highly expressed in both the heart and kidneys and circulating miR-21 can serve as a diagnostic and prognostic marker in CRS2. miR-21 has been associated with poor prognosis in most primary organ failures, which suggests that inhibition of miR-21 may be a potential therapeutic target for CRS3. Using bioinformatics analysis, Romana Ishrat *et al*. [154] determined that some miRNAs, such as miR-122-5p, miR-222-3p, miR-21-5p, miR-5p, miR-3p, miR-24-3p and miR-143-3p as well as some related target genes including transforming growth factor-β1, X-linked inhibitor of apoptosis protein, Lamin-B2, N-alpha-acetyltransferase 50, Nucleoside diphosphate-linked moiety X motif 3, YME1-like protein 1, Insulin-like growth factor 1 receptor, DEAD box protein 6, Protein argonaute-2, Myc proto-oncogene protein, and Protein Hook homolog 3 *(TGF-β1*, *XIAP*, *LMNB2*, *NAA50*, *NUDT3*, *YME1L1*, *IGF1R*, *DDX6*, *AGO2*, *MYC* and *HOOK3*, may be associated with the pathology of CRS3 and can be potential diagnostic biomarkers and therapeutic targets. This will require further experimental and clinical validations.

### 3.6 Stem Cell Therapy

Stem cells have the potential to treat many diseases in regenerative medicine due to their self-renewal and multi-directional differentiation potential. Stem cells function through paracrine mechanisms, modulating apoptosis, reducing oxidative stress and inflammatory mediators, improving damaged tissue and inducing a favorable remodeling environment for organs. These favorable qualities has been demonstrated in experimental models of acute and chronic kidney injury [155, 156, 157]. A recent study reported that adipose-derived stem cells (ADSCs) can alleviate the pathophysiological changes of CRS3 [158]. The results of this study demonstrated that the levels of inflammatory factors, such as serum Interferon-γ (IFN-γ), TNF-α, IL-1β, IL-1α, IL-6 and interleukin-10 (IL-10) levels, were significantly increased in the CRS3 murine model. Abnormal elevation of these inflammatory factors leads to cardiac dysfunction, which is characterized by decreased left ventricular fractional shortening and increased left ventricular end-diastolic and end-systolic volumes. This study also reported the advantages of ADSCs in the treatment of CRS3.

In comparison with bone marrow-derived mesenchymal stem cells (MSCs), which must be obtained through minimally invasive surgery, ADSCs have many advantages. For example, ADSCs can be obtained from adipose tissues, which are more abundant than bone marrow stem cells, easier to culture, and faster to grow. Although stem cells have favorable therapeutic prospects in the treatment of CRS3, there are still many limitations in their clinical usage, such as the complexity of stem cell sources, the safety and effectiveness of stem cell therapy due to the uncontrolled preparation process and quality control of stem cells. In addition, stem cells may be potentially carcinogenic, which greatly limits their applications in clinical medicine. There is emerging evidence that MSC-exosomes can serve as natural carriers for targeted drug delivery. Therefore, therapeutic drugs can be efficiently incorporated into exosomes and then delivered to damaged tissues. In addition, MSC exosomes also comprise bioactive substances such as proteins, mRNAs and miRNAs. Studies have reported MSC-exosomes in AKI [159], and it is also a promising method for cell-free treatment of AKI and CRS3. These studies have demonstrated that MSC-exosomes can improve kidney and heart damage, implying that MSC-exosomes are a promising cell-free therapy for CRS3.

### 3.7 Nanodrugs

There is evidence that the burst of active oxygen and reactive nitrogen (RONS) are major contributors to the progression of AKI [160, 161]. Because of the complex and unique physiological structure of the kidney, most anti-oxidation and anti-inflammatory small molecule drugs are ineffective due to the lack of specificity to kidney tissue and their side effects [162]. Recent studies, including our own, show that nano drugs can target the kidney to solve the limitations of current AKI treatment by controlling the size, shape and surface characteristics of nano drugs [163]. Nano drugs for AKI mainly include nano-RONS-sacrificial agent, antioxidant nano enzyme, and the nano carrier of antioxidant anti-inflammatory drugs. These nano drugs have demonstrated important therapeutic effects, such as reducing oxidative stress damage, restoring kidney function, and are associated with low adverse effects [162]. Ni *et al*. [164] found that molybdenum-based nanoclusters can be utilized as antioxidants to improve AKI. Zhao *et al*. [165] studied the redox mediated artificial non enzyme antioxidant MXene nano platform to alleviate AKI. Zhang *et al*. [166] developed biodegradable self-assembled ultra-small nano dots as active oxygen/nitrogen species scavenger, which can significantly improve AKI. Yu *et al*. [167] found that cerium dioxide nanoparticles targeting mitochondria with atorvastatin combined with the ROS responsive nano drug delivery system has favorable effects in the treatment of sepsis-induced AKI. Wang *et al*. [163] found that selenium nanoparticles can alleviate AKI via regulating the GPx-1/NLRP3/Caspase-1 pathway. In addition, nano drugs may play a role in treating heart disease. Haley *et al*. [168] discussed the clinical feasibility of the therapeutic strategy of delivering anti-inflammatory drugs to the heart muscle through biodegradable polymers, liposomes, hydrogels and nanoparticles-based drug delivery models (NDDM). NDDM is a promising method to successfully treat ischemic HF by delivering anti-inflammatory agents to the myocardium. Tang *et al*. [169] found that platelet nanobubbles fused with stem cells can target repair of cardiac injury. Collectively, these studies suggest that nano drugs not only can be used to treat AKI, but also to improve CRS.

Compared with traditional drugs, nano drugs possess several advantages in the treatment of AKI. Nanodrugs have a variety of materials, flexible sizes and shapes [162]. In addition, nano drugs can be targeted by molecular modification to locate the lesion site, to better control targeting of specific tissues and organs. Furthermore, nanomaterials can also be used as a drug delivery platform to improve the biocompatibility and stability of drugs, and the controlled release of drugs. However, the difference between species is the most important challenge for the clinical transformation of nano drugs. Most studies on nano drugs are only based on rodent models, and not human AKI disease models [170]. Compared with humans, the capillary density and mitochondrial density of mouse kidney tissue are much higher because the metabolic rate of mice is almost seven times than that of humans. AKI patients have diverse genetic and disease backgrounds, such as diabetes, liver and other diseases [171, 172], which may affect the metabolism and efficacy of nano drugs. Therefore, different animal models are necessary to establish multi-dimensional validation, such as the use of the AKI model of zebrafish for validation [173, 174]. Finally, the biocompatibility and long-term safety of nano drugs are unknown and need to be validated in human studies prior to their use in clinical medicine [175].

### 3.8 Intestinal Microbiota

There is now evidence that intestinal dysbiosis is closely linked to AKI, shedding light on kidney–intestine crosstalk in AKI [176, 177, 178]. Zhu *et al*. [179] demonstrated that the supplementation of Lactobacillus casei Zhang could prevent AKI and impede the progression of CKD by improving intestinal flora, increasing the levels of short chain fatty acids (SCFAs) and nicotinamide in the serum and kidney. Yang *et al*. [176] reported that the increase of Enterobacteriaceae and the decrease of Lactobacillus and Ruminococcus were hallmarks of dysbiosis induced by IRI and were related to the decreased levels of SCFAs, intestinal inflammation, and the leaky gut. They also confirmed that the intestinal microbiota controls the severity of AKI through regulation of the immune system. This renal protective effect is related to the reduction of Th17 and Th1 responses as well as the expansion of regulatory T cells and M2 macrophages [176]. A study by Andrade Oliveira *et al*. [180] also showed that SCFAs derived from intestinal microbiota prevents IRI-AKI, suggesting a crosstalk between kidney and intestine. Lee *et al*. [181] found that Lactobacillus salivarius BP121 prevented cisplatin-induced AKI by inhibiting uremic toxins and alleviating dysbiosis. Metabolites derived from gut microbiota also play an important role in AKI. For example, D-serine derived from gut microbiota can mitigate AKI [182]. Thus, targeting intestinal microbiota may provide a new therapeutic strategy for AKI and CRS3.

### 3.9 Traditional Chinese Medicine (TCM)

A number of studies, including our own, have also confirmed that TCM and active monomers can also improve kidney function in AKI though different mechanisms, including inhibiting inflammation, cell apoptosis, necroptosis, ferroptosis, and decreasing oxidative stress [183, 184]. Our recent study found that Oroxylin A, the main active component of Scutellaria baicalensis, prevented AKI and progression to CKD by inducing PPARα-BNIP3 signaling mediated mitophagy [184]. Sun *et al*. [185] has been shown to improve renal tubular injury induced by IRI in mice through the Keap1/Nrf2/antioxidant response element (ARE) pathway. Peng *et al*. [186] found that Shikonin can attenuate apoptosis, oxidative stress and the inflammatory response of renal tubular epithelial cells in a sepsis-AKI model through the nicotinamide adenine dinucleotide phosphate oxidase 4/phosphatase and tensin homologue deleted on chromosome ten (PTEN) pathway. In addition, many TCM including Astragaloside IV, Alpinetin, Astaxanthin (ATX), Baicalin, Cordyceps sinensis as well as Curcumin have been shown to improve AKI [187]. TCM formula can also improve AKI. A multicenter randomized controlled clinical trial showed that Chuan Huang Fang formula combined with reduced glutathione can be used to treat AKI (1–2 grades) in CKD patients (2-4 stages) [188]. In addition, Zou *et al*. [189] showed that intestinal flora mediates the protective effect of Qiong-Yu-Gao, a TCM formula, on cisplatin induced AKI by increasing the production of SCFA, thereby inhibiting the expression and activity of histone deacetylase, and reducing the accumulation of uremic toxins. However, it is noted that some TCMs, such as aristolochic acids and alkaloids, are deleterious to the kidney [190]. Therefore, the nephrotoxicity of TCM will need to be carefully evaluated before determining its clinical application.

## 4. Discussion

### 4.1 Targeting Mitochondrial Dysfunction in CRS3

Mitochondrial dysfunction, one of the molecular links between the kidneys and heart, plays a crucial role in CRS3. Targeting mitochondrial dysfunction may serve as a therapeutic target to treat kidney and heart disease in CRS3. However, the mechanism for these benefits has not been fully clarified. The underlying mechanisms of inflammatory factors and biomolecules in damaged kidney tissues and their effects on heart tissues after AKI remain elusive. Although several mechanisms are involved in maintaining mitochondrial function, determining which regulatory mechanism possesses better therapeutic effects in CRS3 still require further investigation. Myocardial damage can also be caused by mechanical stress such as calcium metabolism disorders, intracellular acidosis and fluid overload. The relationship between these factors and mitochondrial dysfunction will also need to be clarified.

### 4.2 Crosstalk between Kidney and Heart

In addition to mitochondrial dysfunction, the crosstalk between the kidney and heart is also key in the treatment of CRS3. Mechanistically, the crosstalk between organs after tissue injury may involve soluble mediators and their target receptors, cellular mediators, especially immune cells, as well as newly discovered neural immune connections. Khamissi *et al*. [191] identified kidney-released circulating osteopontin (OPN) as a novel AKI-acute lung injury (ALI) mediator. OPN released from renal tubule cells triggered lung endothelial leakage, inflammation, and respiratory failure [191]. In CRS3, the mechanism of kidney derived inflammatory factors, including IL-1β, IL-6 and other mediators, were reported to cause acute myocardial injury. In the future, analyses based on single cell sequencing and the feasibility of using ligand-receptor analysis will help to discover more links that can mediate the crosstalk between the kidneys and the heart. Targeting these mediators may be a promising therapeutic strategy. Since acute injuries can progress to chronic diseases, timely and reasonable treatments are necessary to prevent this adverse outcome of AKI.

### 4.3 Cardiomyocyte Death in CRS3

Although different modes of cell death have been observed in renal tubular epithelial cells and cardiomyocytes during the progression of AKI and cardiac injury, whether there is a common mechanism of cell death in the kidney and heart remains unknown. Thus, developing convenient, accurate and noninvasive methods to predict the severity of tissue injury and the types of cell death in CRS3 will be necessary to develop therapeutic treatments for CRS3.

### 4.4 Dual Roles of Different Cell Types

The contribution of different cell types to the progression of CRS3 still needs to be investigated. Targeting distinct cell types in kidney or heart tissues may generate different outcomes. For example, promoting mitophagy in vascular smooth muscle cells (VSMCs) advances the progression of atherosclerosis (AS), while in endothelial cells and macrophages, promoting mitophagy is atheroprotective. These controversial results provide further evidence that different cell types playing different roles may determine the progression and fate of certain diseases. Therefore, comprehensive studies of the roles and regulatory mechanisms of different cells in CRS3 will be beneficial for developing new therapeutic targets for treating CRS3.

### 4.5 Combined Therapy and Targeted Therapy

These studies have shown that melatonin combined with other treatments can overcome the shortcomings of a single drug and enhance its efficacy. For example, melatonin combined with mitochondria-targeting drugs or stem cells can achieve an enhanced therapeutic effect. Innovative drug delivery systems such as nanoparticle-loaded drug delivery, extracellular vesicles, and molecular structure optimization to increase bioavailability and tissue targeting will also be required [149, 150].

## 5. Conclusions

The occurrence of AKI is attributed to a rapid deterioration of cardiac function in CRS1, and is responsible for acute cardiac damage in CRS3. Thus, the interaction between the kidney and heart is a bidirectional regulation pattern, which forms a vicious cycle and ultimately contributes to the progression of CRS3. Emerging evidence suggests that mitochondrial and inflammatory factors may be the central link for developing therapeutic targets in CRS3. Targeting mitochondrial dysfunction or inflammatory mediators may serve as a promising therapeutic strategy. We reviewed the existing strategies for CRS3 therapy, including targeting mitochondrial dysfunction, antioxidant, Klotho, melatonin, gene therapy, stem cell therapy, nanodrugs, intestinal microbiota and TCM. In addition, according to the different pathological characteristics of heart and kidney injuries, we propose a combined treatment scheme to overcome the shortcomings of a single factor treatment and enhance the therapeutic efficacy. We also suggest developing more sensitive and accurate non-invasive biomarkers of CRS3 to grade and judge the degree of damage. We also recommend timely and targeted treatment according to the degree of injury and disease progression. However, current studies are mainly based on cell and animal models. Further validation in clinical trials to understand the efficiency and safety of these potential therapeutic strategies are urgently needed.

## Abbreviations

CRS3, Cardiorenal syndrome type 3; AKI, acute renal injury; CKD, Chronic kidney disease; CVD, chronic cardiovascular disease; ROS, reactive oxygen species; SNS, sympathetic nervous system; RAAS, renin-angiotensin-aldosterone system; ${\mathrm{UPR}}^{\text{mt}}$, mitochondrial unfolded protein response; UIRI, unilateral ischemia-reperfusion; I/R, ischemia injury; ICAM-1, intercellular cell adhesion molecule-1; GFR, glomerular filtration rate; ECGs, Hyperkalemic Electro cardio graph Signal; RRT, renal replacement therapy; IS, indole acyl sulfate; PCS, p-cresol sulfate; IAA, indole-3-acetic acid; AHR, aryl hydrocarbon receptor; BI-1, Bax inhibitor-1; Grb2, Growth factor receptor-binding protein 2; ASCs, adipose-derived stem cells; OPN, osteopontin; ALI, acute lung injury; HF, Heart failure; VSMCs, vascular smooth muscle cells; AS, atherosclerosis; TNF-α, tumor necrosis factor-a; IL-1, interleukin-1; IL-6, interleukin-6; IL-17, interleukin-17; IL-12, interleukin-12; IL-10, interleukin-10; IL-1β, interleukin-1β; IL-1α, interleukin-1α; TGF-β1, Transforming growth factor-β1; IFN-γ, Interferon γ; Cox-2, cyclooxygenase-2; FGF-23, fibroblast growth factor-23; ATP, adenosine triphosphate; HMGB1, high mobility group box 1; iNOS, inducible nitric oxide synthase; NO, nitric oxide; NOS, nitric oxide synthase; MSCs, mesenchymal stromal cells; MitoQ, Mitoquinone; SkQ1, 10-(6′-plastoquinonyl) decyltriphenyl phosphonium; AGO2, Quantifying Argonaute 2; DDX6, DEAD-box helicase 6; HOOK3, hook micro-tubule tethering protein 3; MYC, myelocytomatosis oncogene; NOX, The NADPH oxidase; IGF1R, IGF-I receptor; YME1L1, YME1 like 1 ATPase; NUDT3, Nudix protein3; NAA50, N-terminal acetyltransferase 50; IP3R-MCU, Inositol 1,4,5-trisphosphate receptor-Mitochondrial calcium uniporter; Nrf2/Slc7a11, Nuclear factor erythroid 2-related factor 2/Solute carrier family 7 member 11; LMNB2, Lamin B2; ADSCs, Adipose tissue-derived stem cells; PBUTs, protein-bound uremic toxins; FUNDC1, FUN14 domain-containing protein 1; SOD, superoxide dismutase; SCFAs, Short chain fatty acids; ATX, Astaxanthin; TCM, Traditional Chinese Medicine; IL-18, interleukin-18; NLRP6, Nod-like receptor pyrin domain-containing protein 6; ASC, apoptosis-associated speck-like protein containing a caspase-recruitment domain; NLRP3, Nod-like receptor pyrin domain-containing protein 3.
